# The Call to the Medical Profession

**Published:** 1915-04-24

**Authors:** 


### The Hospital

The Workers' Newspaper of Administrative Medicine and Institutional Life, Administration, National Insurance and Health.

No. 1505, Vol. LVIII. SATURDAY, APRIL 24, 1915. PRICE ONE PENNY

### THE CALL TO THE MEDICAL PROFESSION.

Let there be no mistake about the matter; the medical profession is at this moment face to face with an issue greater by far than can be recalled in living memory. The summons is to a service which can only be rendered at the cost of individual sacrifice, and will not be made effective save by united effort. The cause is that of the nation, and it will not brook delay. It is for the profession to rise to the occasion and add to its already considerable efforts the further duty which is asked at its hands. We have heard complaints that men are not forthcoming, and that munitions are not forthcoming; in the course of the next few weeks we are likely, unless the situation is taken promptly and effectively in hand, to hear that doctors are not forthcoming. The reputation of the profession is at stake, as is also the welfare of our new Armies; and leading and organisation are essential to meet the crisis. It is impossible to overstate the seriousness and importance of the occasion, and the response to the official appeal is awaited with anxiety in the highest quarters. What is wanted immediately is large numbers of the younger members of the profession ready to take service in the R.A.M.C., and to accompany the new Armies which are shortly to be sent into the fighting line. To state the exact figures is not permitted, but it may be safely said that they must be expressed in higher terms than hundreds. Unless they are obtained, troops otherwise ready for the field will want an essential part of their equipment, and must either remain at home or go on active service without the protection of an adequate number of medical officers.

Clearly the essential feature of the situation as a matter of organisation is to set free the younger men from the demands of civil practice, while securing the satisfaction of these demands from other sources. The call is for men who are so fortunate as to be less than forty years of age. Some of these have more or less recently qualified and are engaged in hospital or other institutional work. But many—probably the majority—in view of the numerous juniors who have already joined the Army, are, and have been for a greater or less number of years, in practice on their own account. In most cases, too, they have given hostages to fortune in the shape of wives and children, and in any event they have their future means of livelihood to consider. To ask men so situated to put aside the claims of home and work, and to risk the loss of income and position, is a demand which can only be justified by the existence of a great national emergency. This emergency, we are told on the highest authority, is at our very doors, and therefore in one way or another the demand has to be met. Sacrifices are called for not only in the interests of the reputation of the profession, but in the interests of national safety. That they will be made we do not doubt, but in order that they may be made on a sufficiently extensive scale they must be the fruit, not of individual, but of collective effort.

Here, then, is the question: How can the younger members of the profession be set free to accompany the new Armies in the field? Manifestly this can only be effected by senior men accepting wider and more numerous duties, for the civil population has needs which cannot be neglected. In regard to juniors engaged in hospitals or holding other institutional appointments, there ought to be no great difficulty. To some extent the position can be helped by the further appointment of women house physicians and house surgeons, and there will be a certain number of young men who are physically unfit for military service. But these will by no means fully meet the situation, and it seems to us a plain duty of the visiting staffs to take up in turn the duties of resident officers and to combine to carry on the hospitals, even though at a great personal inconvenience, without junior assistants. It will not be creditable to any hospital to detain the very men who are urgently wanted for the military service of the nation.

A greater difficulty is presented by men already settled in practice, and it is to these that the appeal made by the authorities has its most serious message. Manifestly, it is an imperative duty that so far as possible their interests during their absence shall be protected, and this can only be effected by the good will and assistance of the neighbouring practitioners. Such loyal co-operation and good faith are no new things in the profession, and at the present juncture they are paid to the nation as much as to the individual. But we think something more is required. There are men in leading positions in the profession who cannot help in the manner just described. But if they cannot contribute help they can contribute money. The pay offered by the Army is relatively small, and many of those who accept it will accept it with financial anxieties and loss. This loss ought to be distributed over the profession as a whole, if, as is at present true, it will not be met by the State. The time is not one for arguments or bargains. The call of the nation must be answered. Let it be answered by a combined effort in which the active services of the younger men are conspicuously supported by the sacrifices of those condemned to more passive duties.

